# Unraveling the reality of interpersonal violence against children and adolescents in Brazil: a systematic review

**DOI:** 10.1590/0102-311XEN145924

**Published:** 2025-03-31

**Authors:** Clovis Wanzinack, Tainá Ribas Mélo

**Affiliations:** 1 Universidade Federal do Paraná, Curitiba, Brasil.; 2 Universidade Regional de Blumenau, Blumenau, Brasil.; 3 Prefeitura Municipal de Paranaguá, Paranaguá, Brasil.; 4 Centro Universitário Campos de Andrade, Paranaguá, Brasil.

**Keywords:** Violence, Child, Adolescent, Reporting, Violência, Criança, Adolescente, Notificação, Violencia, Niño, Adolescente, Notificación

## Abstract

This study aimed to review scientific publications on reports of violence against children and adolescents in Brazil from 2018 to 2022 based on a systematic literature review following the PRISMA guidelines. Selection encompassed quantitative and qualitative studies published in Portuguese, English, and Spanish from the PubMed Central, LILACS, and SciELO databases. Two trilingual reviewers analyzed the studies according to the eligibility criteria, and the quality of the studies was assessed using the *Checklist for Analytical Cross-Sectional Studies* by the Joanna Briggs Institute. The 21 eligible studies were then analyzed, and the social determinants of health listed were grouped into categories (territory, race/ethnicity, gender, age, type of violence, drugs, perpetrator, and where the act occurred) to create a narrative synthesis about each one. The results show worrisome patterns of ethnic-racial inequalities regarding violence, pointing towards a greater vulnerability of the black population. Analyses based on gender, age, and type of violence also reveal a particular vulnerability of girls, especially regarding sexual violence. The perpetrators were mainly identified as the victims’ fathers and mothers, highlighting the relevance of the family setting in enabling violent acts and showing the need for interventions focused on this context. The underreporting of cases indicates the importance of improving report mechanisms and raising community awareness to ensure that the reality of violence against children and adolescents is presented accurately.

## Abstract (pt)

O estudo teve como objetivo revisar as publicações científicas sobre os relatos de violência contra crianças e adolescentes no Brasil de 2018 a 2022. Isso foi realizado por meio de uma revisão sistemática da literatura que seguiu a diretriz PRISMA. A seleção abrangeu estudos quantitativos e qualitativos publicados em português, inglês e espanhol, provenientes de bases de dados como PubMed Central, LILACS e SciELO. Os estudos foram analisados por dois revisores trilíngues, de acordo com os critérios de elegibilidade. A qualidade dos estudos foi avaliada com base na *Lista de Verificação para Estudos Transversais Analíticos* do Instituto Joanna Briggs. Os 21 estudos elegíveis foram então analisados e os determinantes sociais da saúde listados foram agrupados em categorias para criar uma síntese narrativa sobre cada um. Essas categorias incluíram território, cor de pele/etnia, gênero, idade, tipo de violência, drogas, agressor e local do ocorrido. Os resultados mostram padrões preocupantes de desigualdades étnicas em relação à violência, indicando uma maior vulnerabilidade da população negra. As análises baseadas em gênero, idade e tipo de violência também destacam uma vulnerabilidade particular das meninas, especialmente em relação à violência sexual. Os agressores, principalmente identificados como pais e mães das vítimas, destacam a relevância do ambiente familiar como facilitador da ocorrência de atos violentos, evidenciando assim a necessidade de intervenções focadas nesse contexto. A subnotificação de casos indica a importância de aprimorar os mecanismos de denúncia e aumentar a conscientização comunitária para garantir que a realidade da violência contra crianças e adolescentes seja registrada de forma precisa.

## Abstract (es)

Este estudio tuvo como objetivo revisar las publicaciones científicas sobre informes de violencia contra niños, niñas y adolescentes en Brasil de 2018 a 2022. Se realizó una revisión sistemática de la literatura con base en la directriz PRISMA. La selección incluyó estudios cuantitativos y cualitativos publicados en portugués, inglés y español, de bases de datos como PubMed Central, LILACS y SciELO. Los estudios fueron analizados por dos revisores trilingües según los criterios de elegibilidad. La calidad de los estudios se evaluó en función de la *Lista de Verificación para Estudios Transversales Analíticos* del Instituto Joanna Briggs. Luego, se analizaron los 21 estudios elegibles, y los determinantes sociales de la salud enumerados se agruparon en categorías para crear una síntesis narrativa sobre cada uno. Estas categorías incluyeron territorio, color de piel/etnia, género, edad, tipo de violencia, drogas, agresor y lugar de ocurrencia. Los resultados muestran patrones preocupantes de desigualdades étnicas en relación con la violencia, e indica una mayor vulnerabilidad de la población negra. Los análisis basados en el género, en la edad y en el tipo de violencia también destacan una vulnerabilidad particular de las niñas, especialmente en relación con la violencia sexual. Los agresores fueron, principalmente, padres y madres de las víctimas, lo que destaca la relevancia del entorno familiar como facilitador de la ocurrencia de hechos violentos, así se resalta la necesidad de intervenciones dirigidas a este contexto. El subregistro de casos muestra que es necesario mejorar los mecanismos de denuncia y aumentar la concienciación de la comunidad para que se registre, con precisión, la realidad de la violencia contra los niños, niñas y adolescentes.

## Introduction

The National Policy for Reducing Morbidity and Mortality from Accidents and Violence, implemented by the Brazilian Ministry of Health in 2001, is an important milestone in the fight against violence in the country ^1^. It established how sectors coordinate with each other and internally to provide care in the face of violent incidents, with violence reports being one of the priorities.

Reporting violence against children and adolescents to competent authorities was already mandatory based on the *Statute of the Child and Adolescent* (ECA, acronym in Portuguese) in 1990 ^2^. Only in 2001, through *Ordinance n. 1,968/2001* ^3^, that the report of suspected or confirmed cases of abuse against children and adolescents cared for in the Brazilian Unified National Health System (SUS, acronym in Portuguese) became mandatory.

Reporting is carried out via *Domestic, Sexual, and/or Other Violence Report/Investigation Form* (FNI, acronym in Portuguese), which is filled out at health services. The data is then entered, consolidated, sent to the Violence and Accident Surveillance System (VIVA, acronym in Portuguese) and subsequently sent to Child Protective Services. This system became part of the Brazilian Information System for Notificable Diseases (SINAN, acronym in Portuguese) in 2008 ^1^.

Although underreporting is still present, since not all cases of violence reach health services ^4^, VIVA and SINAN represent advances in terms of case surveillance, as researchers have used these data to characterize violence against children and adolescents in Brazil, identifying an increase in reports over the years ^5^ ^,^ ^6^. This indicates health professionals have more commonly used this public policy to address this issue.

Considering that cases of violence have been systematically reported for 15 years, this study aimed to review scientific publications available on the reported rates of violence against children and adolescents in Brazil from 2018 to 2022.

## Methodology

The protocol for this systematic review was registered in the International Prospective Register of Systematic Reviews (PROSPERO; CRD42023473241) from the University of York (United Kingdom). It was conducted and presented following the *Preferred Reporting Items for Systematic Reviews and Meta-Analyses* (PRISMA).

### Search

The search was carried out in the following databases: PubMed Central, LILACS, and SciELO. Box 1 presents the Boolean descriptors and operators used. Health Science Descriptors (http://decs.bvs.br/) developed by the Latin American and Caribbean Center on Health Sciences Information (BIREME) were adopted.

**Box 1:** Adopted Boolean descriptors and operators.

LANGUAGE	DESCRIPTORS AND BOOLEAN OPERATORS
Portuguese	“violência” AND “criança” AND “adolescente” AND “notificação”
English	“violence” AND “child” AND “adolescent” AND “notification”
Using *	“violência*” AND “crianç*” AND “adolescente*” AND “notificação” “violenc*” AND “child*” AND “adolescent*” AND “notification*”

Source: prepared by the authors.

The Rayyan platform (https://www.rayyan.ai/) was used to identify duplicate articles from the databases used and for the reviewers to blindly include and exclude studies. In case of disagreement, a third reviewer was consulted.

### Types of studies selected

Studies that analyzed violence reports in Brazil and/or discussed their associations with social determinants of health (SDH) were included. The eligibility criteria were: (i) quantitative studies on reports of violence against children and adolescents in Brazil; (ii) studies whose methods included primary or secondary data gathered by SINAN; (iii) published from 2018 to December 2022; (iv) in English, Spanish, or Portuguese; (v) with analysis of violence indicators, SDH, and/or associated factors.

The meaning of “adolescents” used herein follows the criteria adopted by the World Health Organization (WHO), which defines adolescence as the phase between 10 and 19 years of age (young adolescents are aged 10-14 years old; older adolescents are aged 15-19 years old), whereas childhood is the phase from 0 to 9 years old. The period of 2018 to 2022 was chosen to prioritize the analysis of recent trends with consolidated data, since current data better reflect the present situation and facilitate the observation of more immediate changes in public policies and socioeconomic contexts.

Our criteria excluded literature reviews, opinion articles, case studies, editorials, policies, unconventional literature, or conference abstracts (“grey literature”).

### Article tracking and selection

**Figure 1:**
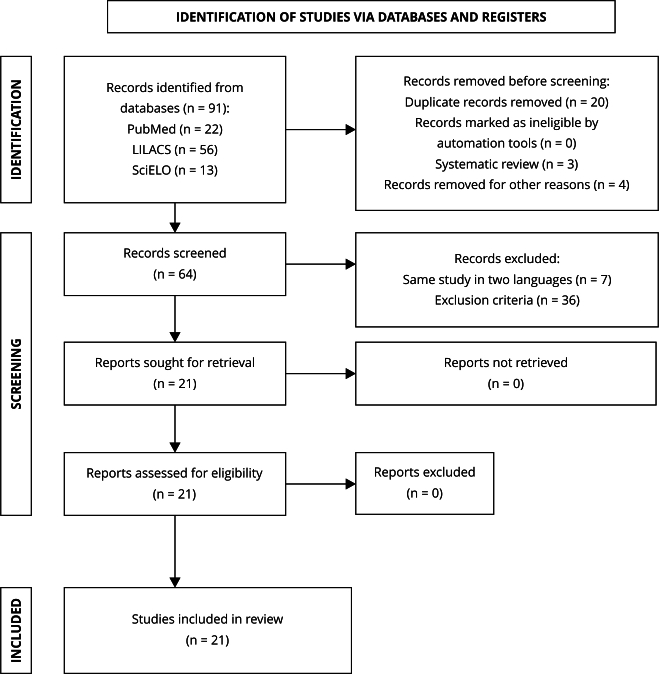
Flowchart showing the screening and selection process of articles based on the PRISMA protocol.

Source: prepared by the authors, based on the PRISMA flowchart (http://prisma-statement.org/).

Two authors performed a double-blind review. Abstracts were screened independently based on our search strategy (Box 1), and disagreements were addressed with the support of a third evaluator (Ph.D.). As aforementioned, the reasons for excluding each article were recorded. Then, abstracts of studies that met the eligibility criteria were read in full (Figure 1).

### Critical evaluation

All selected studies are observational, either cross-sectional or time series analyses, which can be interpreted as several cross-sectional studies. Thus, the quality of the articles included in this systematic review was assessed based on the *Checklist for Analytical Cross-Sectional Studies* by the Joanna Briggs Institute (JBI; https://jbi.global/critical-appraisal-tools).

Each study was appraised according to specific checklist items ^7^: (i) sample inclusion criteria; (ii) level of detail of the subjects and the setting; (iii) level of validity and reliability with which the exposure was measured; (iv) criteria objectivity; (v) identification of confounding factors; (vi) strategies used to deal with confounding factors; (vii) validity and reliability of the outcomes; and (viii) appropriateness of the statistical analysis used.

Eligible studies were classified according to the evidence quality: low for a total score < 50%, moderate between 50% and 74%, or high for a total score ranging 75% to 100%.

### Study management and storage

The articles were stored and managed in the online version of Rayyan as follows: (i) reading the title and abstract of all articles identified based on the search strategy; (ii) excluding duplicates and files in other formats; (iii) comparing the two lists generated by independent researchers; (iv) discussing with these two authors alongside a third evaluator to reach consensus; (v) reading the full versions and performing a critical appraisal of the remaining articles.

### Data collection and synthesis strategy

The data collected included title, authors, journal, year of publication, objectives, study design, measurements, and outcomes, including quantitative and qualitative analysis of SDH, which were grouped into eight categories: territory; race/ethnicity; gender; age; type of violence; drugs; perpetrator; and where the act occurred. The results were presented in a narrative synthesis ^8^ that categorized the findings according to the most common SDH, and a narrative analysis of each category was performed. As a mere description of the studies is insufficient for a narrative synthesis, a textual approach was adopted, in order to analyze implicit associations in each study and between studies, as well as an overall appraisal of the validity of the evidence ^8^. Also, a box were created to provide a descriptive summary of the results and explain the characteristics of this study according to international guidelines for systematic review reports.

## Results and discussion

After full-reading the 21 articles included in this review, we performed a narrative synthesis of the results, categorizing the findings according to the following SDH: territory; race/ethnicity; gender; age; type of violence; drugs; perpetrator; and where the act occurred. The following sections will cover all SDH.

Box 2 shows the description of the articles and their corresponding objectives and SDH. Among the 21 articles selected, 20 were written in Portuguese, representing 95.2% of the total. Of these, 14 articles (66.7%) were also in English, and only one article (4.8%) was also in Spanish. This linguistic distribution reflects the amount of studies in these different languages within the scope of this research.

**Box 2:** Studies included in the systematic review according to year, author(s), title, goals, and social determinants of health (SDH).

STUDY (YEAR)	OBJECTIVE	SDH	CRITICAL APPRAISAL
Santos et al. ^9^ (2018)	To describe reported sexual violence against children and adolescents at school in Brazil during 2010-2014	Territory (Brazil); Race/ethnicity; Gender; Age group; Type of violence; Drugs; Perpetrator; Place of occurrence	High quality
Platt et al. ^18^ (2018)	The scope of this study was to identify the characteristics of sexual abuse against children including the profiles of the victims and the perpetrators, and associated factors notified in a reference health service with the database of the Brazilian Case Registry Database, in a city in Southern Brazil	Territory (Florianópolis); Race/ethnicity; Gender; Age group; Type of violence; Drugs; Perpetrator; Place of occurrence	High quality
Honorato et al. ^25^ (2018)	The article aims to trace a profile of reported child violence in the western region of the state of Pará, with emphasis on physical and sexual violence	Territory (Pará); Race/ethnicity; Gender; Age group; Type of violence; Drugs; Perpetrator; Place of occurrence	Moderate quality
Aguiar et al. ^16^ (2019)	To identify the epidemiological profile of violence against children and adolescents from 2012 to 2016 in the state of Paraná	Territory (Paraná); Race/ethnicity; Gender; Age group; Type of violence; Perpetrator; Place of occurrence	Moderate quality
Abreu et al. ^15^ (2019)	To analyze the correlation between raw rates of rape and year of occurrence, and spatial patterns of notified cases of rape against adolescents	Territory (Pernambuco); Race/ethnicity; Gender; Age group; Type of violence; Drugs; Perpetrator; Place of occurrence	High quality
Oliveira et al. ^6^ (2020)	To describe the epidemiological profile of sexual violence in children and adolescents living in the city of São Paulo	Territory (São Paulo); Race/ethnicity; Gender; Age group; Type of violence; Perpetrator; Place of occurrence	Moderate quality
Oliveira et al. ^27^ (2020)	To describe cases of violence against children and adolescents and completeness of notification forms registered on the SINAN, Manaus, Amazonas State, 2009-2016	Territory (Manaus); Race/ethnicity; Gender; Age group; Type of violence; Drugs; Perpetrator; Place of occurrence	High quality
Pereira et al. ^10^ (2020)	This study aims to characterize the profile of violence victims and likely perpetrators of violence against adolescents, as well as to describe the percentage of notifying municipalities according to the Federation Unit	Territory (Brazil); Race/ethnicity; Gender; Age group; Type of violence; Drugs; Perpetrator; Place of occurrence	High quality
Pinto et al. ^11^ (2020)	To describe the notifications of interpersonal and self-inflicted firearm violence in adolescents and to identify the factors associated with the notification of this event	Territory (Brazil); Race/ethnicity; Gender; Age group; Type of violence; Perpetrator; Place of occurrence	High quality
Dornelles et al. ^21^ (2021)	To characterize violence notified against children in the city of Porto Alegre, Rio Grande do Sul State	Territory (Porto Alegre); Race/ethnicity; Gender; Age group; Type of violence; Drugs; Perpetrator; Place of occurrence	High quality
Salazar López et al. ^12^ (2021)	To describe the characteristics of violence against adolescents in Brazil reported from the VIVA	Territory (Brazil); Race/ethnicity; Gender; Age group; Type of violence; Drugs; Perpetrator; Place of occurrence	High quality
Oliveira et al. ^26^ (2021)	The aim of this study was to identify the epidemiological profiles of violence against children, victims, and their aggressors, and their correlations between socioeconomic and demographic factors analyzed before and during the COVID-19 pandemic	Territory (São Paulo); Race/ethnicity; Gender; Age group; Type of violence; Drugs; Perpetrator; Place of occurrence	High quality
Figueiredo et al. ^22^ (2021)	This study outlines the sociodemographic profile of children, adolescents, women and older adults victim of violence in the city of Porto Alegre, from January 2017 to December 2019	Territory (Porto Alegre); Race/ethnicity; Gender; Age group; Type of violence; Drugs; Perpetrator; Place of occurrence	Moderate quality
Cargnin at al. ^23^ (2021)	To characterize sexual violence cases suffered by women notified by the SINAN in the city of Rio Branco from 2011 to 2016	Territory (Rio Branco); Race/ethnicity; Gender; Age group; Type of violence; Drugs; Perpetrator; Place of occurrence	High quality
Soares et al. ^13^ (2021)	The scope of this study was to analyze the trend, spatialization and circumstances associated with violence against vulnerable populations in Brazil between 2009 and 2017	Territory (Brazil); Race/ethnicity; Gender; Age group; Type of violence; Drugs; Perpetrator; Place of occurrence	High quality
Oliveira et al. ^24^ (2021)	To characterize the epidemiological profile of violence against children and adolescents in the city of Caxias, Maranhão State	Territory (Caxias); Race/ethnicity; Gender; Age group; Type of violence; Drugs; Perpetrator; Place of occurrence	Moderate quality
Oliveira et al. ^28^ (2022)	To describe the characteristics of reported cases of child labor in general and to compare official reported child labor data with data on sexual exploitation and occupational accidents involving children and adolescents between 2017 and 2021	Territory (Amazonas); Race/ethnicity; Gender; Age group; Type of violence	High quality
Platt et al. ^17^ (2022)	To evaluate the completeness, consistency and duplicity of records of child sexual abuse on the SINAN in Santa Catarina, between 2009 and 2019	Territory (Santa Catarina); Race/ethnicity; Gender; Age group; Type of violence; Drugs; Perpetrator; Place of occurrence	High quality
Leite et al. ^19^ (2022)	To identify the frequency of negligence against girls and women in Espírito Santo, and its association with the characteristics of the victim, the aggressor, and the aggression	Territory (Espírito Santo); Race/ethnicity; Gender; Age group; Type of violence; Drugs; Perpetrator; Place of occurrence	High quality
Riba & Zioni ^14^ (2022)	The present study is characterized as a descriptive epidemiological study of time-series, based on secondary data obtained from the SINAN-NET and TabWin	Territory (Brazil); Race/ethnicity; Gender; Age group; Type of violence; Perpetrator; Place of occurrence	Moderate quality
Leite et al. ^20^ (2022)	To identify the frequency of reported cases of recurring violence against adolescents and their association with victim, violence, and aggressor characteristics	Territory (Espírito Santo); Race/ethnicity; Gender; Age group; Type of violence; Drugs; Perpetrator; Place of occurrence	High quality

SINAN: Brazilian Information System for Notificable Diseases; VIVA: Violence and Accident Surveillance System.

Source: prepared by the authors.

The critical analysis showed that most studies (15 out of 21) fully met the criteria established in the appraisal instruments, representing approximately 71.4% of the total sample. Thus, these studies were rated as “high quality”. Around 28.6% of the articles were considered of moderate quality. Notably, none of the articles received the lowest possible rating, justifying the absence of low-quality studies in the sample.

### Territory

Territory was present in all studies, whether regionally or nationwide, confirming the need for territorialization to understand the phenomenon of violence.

In this sense, this research analyzed 21 studies that address the issue in different territories and periods. The sections on time and geography in this article provide a comprehensive view of violence against children and adolescents in different parts of Brazil, enabling the identification of trends and patterns over time and in different regions.

This time frame facilitates the analysis of changes over the years, identifying possible advances or variations in violence rates.

The research approach includes different geographic scopes, including the whole country ^9^ ^,^ ^10^ ^,^ ^11^ ^,^ ^12^ ^,^ ^13^ ^,^ ^14^ and specific states and municipalities. This enabled a more detailed analysis, identifying specific variations in violence rates at different territorial scales.

The research encompassed several Brazilian states, including Pernambuco ^15^, Paraná ^16^, Santa Catarina ^17^ ^,^ ^18^, Espírito Santo ^19^ ^,^ ^20^, Rio Grande do Sul ^21^ ^,^ ^22^, Acre ^23^, Maranhão ^24^, Pará ^25^, São Paulo ^6^ ^,^ ^26^, and Amazonas ^27^ ^,^ ^28^. This geographic diversity enabled a better understanding of violence considering potential regional differences.

Some locations, such as Porto Alegre (Rio Grande do Sul State) ^21^ ^,^ ^22^ and Espírito Santo ^19^ ^,^ ^20^, appear in different studies with varying time frames. Thus, data on the same region can be compared over time, contributing to understanding alterations and consistency in violence rates.

This research not only encompasses a variety of states but also specific mesoregions and municipalities, such as Florianópolis (Santa Catarina State) ^18^, Manaus (Amazonas State) ^27^, Porto Alegre ^21^ ^,^ ^22^, Rio Branco (Acre State) ^23^, Caxias (Maranhão State) ^24^, and São Paulo ^6^ ^,^ ^26^. Furthermore, the mesoregion of Lower Amazon ^25^ in the state of Pará, and the mesoregion of the state of Amazonas ^28^ are also included. This analysis can reveal critical nuances specific to different regions that might otherwise go unnoticed in a broader approach.

### Race/ethnicity

Studies conducted in different periods and regions of Brazil reveal striking patterns of violence that reflect deeply ingrained ethnic-racial inequalities. From 2011 to 2017 ^11^, cases of violence with firearms increased, particularly among black individuals, highlighting a disturbing connection between mortality rates and violent incidents with firearms. These patterns are aggravated by the continued lack of opportunities and access to education for the black population, culminating in lower educational levels.

In the state of Paraná ^16^, from 2012 to 2016, despite the initial prevalence of violence towards the white population, a more in-depth analysis demonstrated that the impacts of such incidents were more pronounced in the black population, especially among black girls aged 0 to 4 years, followed by the Indigenous population. These results highlight the need for specific protection strategies geared towards these ethnic-racial groups.

The predominance of female and black victims nationwide from 2010 to 2014 ^18^ is a confirmation of the significant social disparities between these and other groups, especially among children aged 0 to 9 and adolescents aged 10 to 19. These data underscore the urgency of developing sensitive approaches that consider social, racial, and gender intersections when designing strategies to prevent and fight violence.

In São Paulo ^6^, between 2015 and 2017, children aged 5 to 9 were the most vulnerable to sexual violence, particularly girls; the incidence is also higher among black people. This reinforces the pressing need for protective strategies for this vulnerable age group, particularly considering the intersection between gender and race.

In Espírito Santo ^20^, from 2011 to 2018, cases of neglect were concerningly prominent among girls aged 0 to 9 and older women, especially black and mixed-race women, highlighting the importance of specific policies for vulnerable ethnic-racial groups.

Analyses regarding the period from 2009 to 2019 ^14^ revealed that physical domestic violence in Brazil had a more significant impact on children aged 0 to 4, especially among Indigenous and mixed-race people, signaling the urgent need to protect these vulnerable ethnic groups.

In the state of Amazonas ^28^, between 2017 and 2021, mixed-race people were the most prevalent victims of sexual exploitation, and most work accidents involved male and mixed-race individuals.

In Manaus ^27^, from 2009 to 2016, adolescents aged 10 to 14, mainly mixed-race and female, were most affected by violence, highlighting the urgency of specific protective measures for this age group, especially considering the racial aspect.

In Rio Branco ^23^, between 2011 and 2016, a significant increase in cases was observed, mainly during 2012 and for young adolescents aged 10 to 14, single, and mixed-race, thus showing the pressing need for specific protection strategies for this ethnic-racial group.

These studies reflect a myriad of challenges from different Brazilian regions, highlighting the urgency for specific instructions and targeted policies to protect vulnerable ethnic-racial groups and reduce these types of violence.

### Gender, age, and type of violence

Over different periods and regions in Brazil, several studies have revealed alarming information about patterns, risks, and vulnerabilities related to violence toward children and adolescents.

For example, from 2011 to 2017 ^11^, the data reveal a worrying panorama in Brazil: 59.05% of cases of violence involving firearms affected boys aged 10 to 14, while sexual violence, often combined with physical violence, affected 47.7% of girls in the same age group.

From 2010 to 2014 ^9^, sexual violence reports in Brazilian schools predominantly included female victims, amounting to 63.8% of cases. Rape was the most common type of violence, present in 60.9% of incidents.

In Brazil, from 2009 to 2016 ^12^, there was a progressive increase in reported cases of violence against adolescents, especially physical violence, which is more prevalent for boys under 9 years of age. However, research indicates a greater risk of sexual violence for girls, regardless of age.

In Porto Alegre ^22^, from 2017 to 2019, there were 8,394 reports of violence towards children and adolescents, with 78.2% of incidents involving female victims. Negligence and sexual violence were the most recurrent types. In another study in Porto Alegre ^21^, in 2017, sexual violence was more prevalent among girls (63%), while neglect was more prominent among boys (53%). Furthermore, children aged from 0 to 5 were more susceptible to neglect.

In the state of Paraná ^16^, from 2012 to 2016, there was a predominance of violent incidents among females, representing 56.24% of reported cases. Negligence and physical, psychological, moral, and sexual violence were the most prevalent types, with a gradual increase in reports over these years.

Another worrying trend was the 662.5% increase in the number of reports on child sexual violence in Santa Catarina ^17^ from 2009 to 2019.

In Espírito Santo ^19^, from 2011 to 2018, negligence predominantly affected the 0-9 age group, accounting for 55% of the records. Alarmingly, girls in this age group were subjected to negligence 108.67 times more often than women aged 20 to 59, who also faced neglect.

In the state of Amazonas ^28^, from 2017 to 2021, the predominance of child labor and sexual exploitation reinforces the vulnerability of children and adolescents, especially females.

These data highlight the urgent need for effective measures to protect children and adolescents, and the underreporting of such cases also indicates that the reality may be more severe than the records indicate.

### Alcohol, perpetrator, and where the act occurred

From 2009 to 2019 ^14^, Brazil reported an alarming total of 118,499 cases of violence against children and adolescents, revealing a worrying scenario of aggressions that deserve immediate attention. Physical violence, mainly perpetrated by the father (41.13%) and mother (39.84%), occurs primarily in the family setting (81.1% of reported cases).

From 2010 to 2014 ^18^, 1,546 cases of sexual violence against children aged 0 to 9 were reported throughout Brazil in school settings. A significant portion of these cases (31.8%) were repeated offenses; in 85.5% of the incidents, the perpetrators were male, and in 46.9% of the episodes, the victims knew the perpetrator.

In 2017, 5,308 cases of violence against children and young adolescents up to 12 years old were recorded in Porto Alegre ^21^. More than a third of these episodes were recurrent (36%), and 19% of cases involved more than one perpetrator. Most perpetrators (68%) were male, and approximately 35% were under the influence of alcohol at the time of the aggression. A worrying finding is that 72% of incidents occurred in the victims’ homes, and in 62% of the cases, the perpetrators were part of the child’s family.

In the state of Paraná ^16^, between 2012 and 2016, 48,870 cases of violence against children and adolescents were recorded. Aggression also prevailed in the home setting, with mothers (44.6%) and fathers (29.9%) being identified as the main perpetrators.

In Manaus ^27^, between 2009 and 2016, 4,638 children were victims of violence, predominantly sexual violence, with an alarming peak in 2013. Most perpetrators were male (57.2%), and the aggressions occurred predominantly in the victims’ homes (60.1%), where men represented an even more significant majority of perpetrators (80%). In 14.8% of cases, it was suspected that the perpetrators had consumed alcohol.

Finally, in Caxias ^24^, from 2013 to 2014, 383 cases of violence against children and young adolescents up to 14 years old were recorded. Surprisingly, the mothers were the main perpetrators (63.8%). Most of the violence occurred in the victims’ homes (94.3%), with most perpetrators having consumed alcohol (7.2%).

## Final considerations

The included studies encompass both regional and local scales and reveal a detailed overview of violence against children and adolescents in Brazil. The inclusion of several states enables a comprehensive representation of the country’s territorial diversity. This multifaceted approach helps us identify temporal and spatial trends, enriching the understanding of the dynamics of violence over time and in different regions.

The temporal analysis, comparing different periods in specific locations such as Porto Alegre and Espírito Santo, contributes to a deeper perception of variations in violence rates. Detailed exploration at more specific levels, including mesoregions and municipalities, such as Florianópolis, Manaus, Caxias, and Rio Branco, highlights crucial nuances that could go unnoticed in broader analyses, underscoring the importance of considering local specificities when formulating strategies to combat violence.

The results also show worrying patterns of ethnic-racial inequalities regarding violence. Black people are more vulnerable in multiple regions and periods, which shows an intersection between violence, race, and lack of educational opportunities. Thus, there is an urgency for strategies targeted at specific ethnic-racial groups, particularly black children and adolescents, highlighting the need for interventions sensitive to racial inequalities.

Analyses based on gender, age, and type of violence reveal different patterns of vulnerability (particularly girls), especially to sexual violence and in specific age groups. The prevalence of rape in schools points towards the urgent need for specific and gender-sensitive preventive measures.

The perpetrators, mainly identified as the victims’ parents, highlight the relevance of the family setting in allowing violent acts. The home continues to be the main location of violent incidents, emphasizing the urgency of interventions focused on this context. The significant presence of perpetrators under the influence of alcohol shows the complexity of the phenomenon and the need for preventive strategies that address substance use.

Underreporting shows the importance of improving reporting mechanisms and raising community awareness to ensure the data accurately reflects the reality of violence against children and adolescents.

In summary, the results highlight the complexity and severity of violence, emphasizing the need for integrated and context-sensitive strategies to protect children and adolescents throughout Brazil effectively. Understanding territorial and family dynamics, as well as ethnic-racial and gender inequalities, is crucial to guide effective preventive policies and actions.
